# The degree to which the cultural ideal is internalized predicts judgments of male and female physical attractiveness

**DOI:** 10.3389/fpsyg.2022.980277

**Published:** 2022-10-19

**Authors:** Bethany J. Ridley, Piers L. Cornelissen, Nadia Maalin, Sophie Mohamed, Robin S. S. Kramer, Kristofor McCarty, Martin J. Tovée

**Affiliations:** ^1^Department of Psychology, Northumbria University, Newcastle upon Tyne, United Kingdom; ^2^Department of Psychology, Birmingham City University, Birmingham, United Kingdom; ^3^Aberdeen Royal Infirmary, NHS Grampian, Aberdeen, United Kingdom; ^4^School of Psychology, University of Lincoln, Lincoln, United Kingdom

**Keywords:** body ideals, body composition, muscularity, adiposity, attractiveness, body fat

## Abstract

We used attractiveness judgements as a proxy to visualize the ideal female and male body for male and female participants and investigated how individual differences in the internalization of cultural ideals influence these representations. In the first of two studies, male and female participants judged the attractiveness of 242 male and female computer-generated bodies which varied independently in muscle and adipose. This allowed us to map changes in attractiveness across the complete body composition space, revealing single peaks for the attractiveness of both men and women. In the second study, we asked our participants to choose the most attractive male and female bodies in a method of adjustment task in which they could independently vary muscle and adipose to create the most attractive body. We asked whether individual differences in internalization of cultural ideals, drive for muscularity, eating disorder symptomatology and depressive symptoms could systematically shift the location of peak attractiveness in body composition space. We found a clear preference by both genders for a male body with high muscle and low adipose, and a toned, low adipose female body. The degree of internalization of cultural ideals predicted large individual differences in the composition of the most attractive bodies.

## Introduction

The concept of body image refers to a person’s “inside view” of their body, i.e., their feelings, perceptions, thoughts, and beliefs about their body that impact how they behave toward it (Cash, 2004). Body image and appearance is an important concern for most men and women (Olivardia et al., 2004; Kelley et al., 2010; Runfola et al., 2013). While poor body image is linked to the development of a range of psychological and psychiatric problems, including depression and eating disorders (Freeman et al., 1985; Stice, 2002; Liechty, 2010; Junne et al., 2019), positive body image has been found to have unique associations with well-being, self-care and eating behaviors (Avalos et al., 2005; Andrew et al., 2014; Tylka and Wood-Barcalow, 2015). In Western society, much of the blame for poor body image can be attributed to the cultural emphasis on appearance and the importance attached to attractiveness on the one hand, together with the promulgation of idealized media images that place pressure on women to achieve a comparable aesthetic appearance on the other hand (Levine and Murnen, 2009). For several decades, the Western ideal female body has emphasized a low body mass index (BMI; Swami et al., 2010; Crossley et al., 2012). This has become exaggerated in the ultra slim bodies found in fashion models (Tovée et al., 1997; Jestratijevic et al., 2020), and in its most extreme form has manifested in the social media trends of thinspiration and bonespiration (Talbot et al., 2017). More recently, this fixation on body fat has been compounded by the rise of the “athletic” ideal, epitomized by the fitspiration trend on social media, which portrays a body shape that is not only very slender, but also toned and muscular (Benton and Karazsia, 2015; Tiggemann and Zaccardo, 2015, 2018; Boepple and Thompson, 2016; Carrotte et al., 2017; Bozsik et al., 2018). This body type is potentially even harder to achieve than a thin body and can lead to greater dissatisfaction than the previous thin female ideal (Uhlmann et al., 2018), with links to negative psychological outcomes (Cunningham et al., 2019).

The corresponding ideal body for men in Western society is characterized by both a high degree of upper body muscularity (the V-shaped torso) and a low degree of body fat, with the latter enhancing the salience of the former (Maisey et al., 1999; Leit et al., 2002; Cafri et al., 2005; Ridgeway and Tylka, 2005; McCreary, 2007; Murray et al., 2017). The muscular ideal has long been propagated in Western culture as shown by the appearance of male models (Frederick et al., 2005; Lanzieri and Cook, 2013), film stars (Pope et al., 2000a,b) computer game avatars (Martins et al., 2011), and action figures (Baghurst et al., 2006). It is also now further extended by the hyper muscular male, low adipose bodies in fitspiration content posted on social media (Carrotte et al., 2017; Tiggemann and Zaccardo, 2018). After viewing such content, men are more likely to engage in excessive exercise and to take anabolic steroids with potential negative health outcomes (Cafri et al., 2006; Horwitz et al., 2019; Mossman and Pacey, 2019; Tiggemann and Anderberg, 2020).

An important issue to consider is how these ideals are derived and communicated to individuals within a population. The principal sociocultural explanation, the tripartite influence model, emphasizes the importance of social pressure derived from three sources: family, peers, and media (Thompson et al., 1999; Shroff and Thompson, 2006). Individuals will vary both in the extent to which they are exposed to these pressures and also in the extent to which they internalize these pressures and the message they convey (i.e., the extent to which the appearance-related messages are judged to be important and relevant to themselves). It is this internalization, together with social comparison processes, which are proposed to be the link between the societal message of a specific body ideal and the development of body dissatisfaction (Stice et al., 2001; Tiggemann, 2003; Keery et al., 2004; Sypeck et al., 2006; Schaefer et al., 2015). We note that more recent iterations of the tripartite influence model not only incorporate both thin-ideal as well as muscular-ideal internalization, but also attempt to integrate these processes with objectification theory through the agency of body surveillance (Frederick et al., 2022). Body dissatisfaction would thus arise because most people do not match these appearance ideals but feel pressure to conform to this archetype.

From an applied psychological point of view, attractiveness is also an important factor in social interactions, including college admissions and job applications (Shtudiner, 2020; Ong, 2022; Ruffle et al., 2022; Shapir and Shtudiner, 2022). A number of studies have suggested that more attractive people are offer higher salaries in the job market and earn more on average, although there is no evidence that they perform better than colleagues (Heilman and Saruwatari, 1979; Mobius and Rosenblat, 2006). For example, there seems to be a “beauty premium” for both men and women when applying for internships in accountancy firms, with attractive individuals being rated to possess more of the desirable attributes for the position (Shapir and Shtudiner, 2022). Thus, attractiveness judgements can have significant consequences in real word situations.

### The current study

In this study we ask: what does the internal *visual* template of the ideal body for men and women look like? To our knowledge, there have been few attempts to address this question directly [but see, e.g., (Brown and Slaughter, 2011; Mohamed et al., 2021)]. Instead, most evidence appears more indirect. That the thin ideal exists as a cultural phenomenon in Western society is supported by studies of social media content [e.g., (Boepple and Thompson, 2016; Boepple et al., 2016a)] and *post-hoc* ratings of beauty pageant winners (Bozsik et al., 2018). In the verbal domain, for example, Ridgeway and Tylka (2005) used an open-ended interview technique to show that the ideal male body derived from five key components—overall body muscularity, overall body leanness, being tall, V-shape torso, and a muscled abdominal region. Undesirable body characteristics included fat, short stature, and low body fat coupled with low muscle tone leading to small girth [see also (Pope et al., 2000a,b; Grogan and Richards, 2002)]. In the visual domain, there are a number of on-line and laboratory-based studies which have exposed participants to thin and athletic ideal images, which lead to increases in body dissatisfaction. As a specific example, Robinson et al. (2017) searched Google and Instagram using keywords such as “thinspiration,” “athletic fitspiration,” and “muscle fitspiration” to return 85 images. These images were then rated on a 7-point Likert scale for thinness, tone, athleticism, and muscularity, and a subset of them were assigned to one of three experimental conditions—thin ideal, athletic ideal, and muscular ideal—based on these rater statistics. While this study clearly shows that a set of appropriately defined experimental stimuli can have an effect on body dissatisfaction in the predicted direction, it is unclear whether the images that participants were shown *necessarily* align precisely with any individual’s own internal visual representation of a thin/athletic/muscular ideal. Therefore, in this study we aim to visualize directly what an ideal female/male body looks like separately for male and female raters and investigate how individual differences in thin/muscular internalization, for example, may influence it. To do this, we make the assumption that maximally attractive and “ideal” can be treated as synonymous. If so, we can ask participants to rate images showing systematic variation in biometric properties and locate where in this parameter space the most attractive images reside. We can then ask if this process yields images consistent with externally defined ideals, such as the thin ideal or athletic ideal.

### The problem with BMI, WHR and SWR

**Figure 1:**
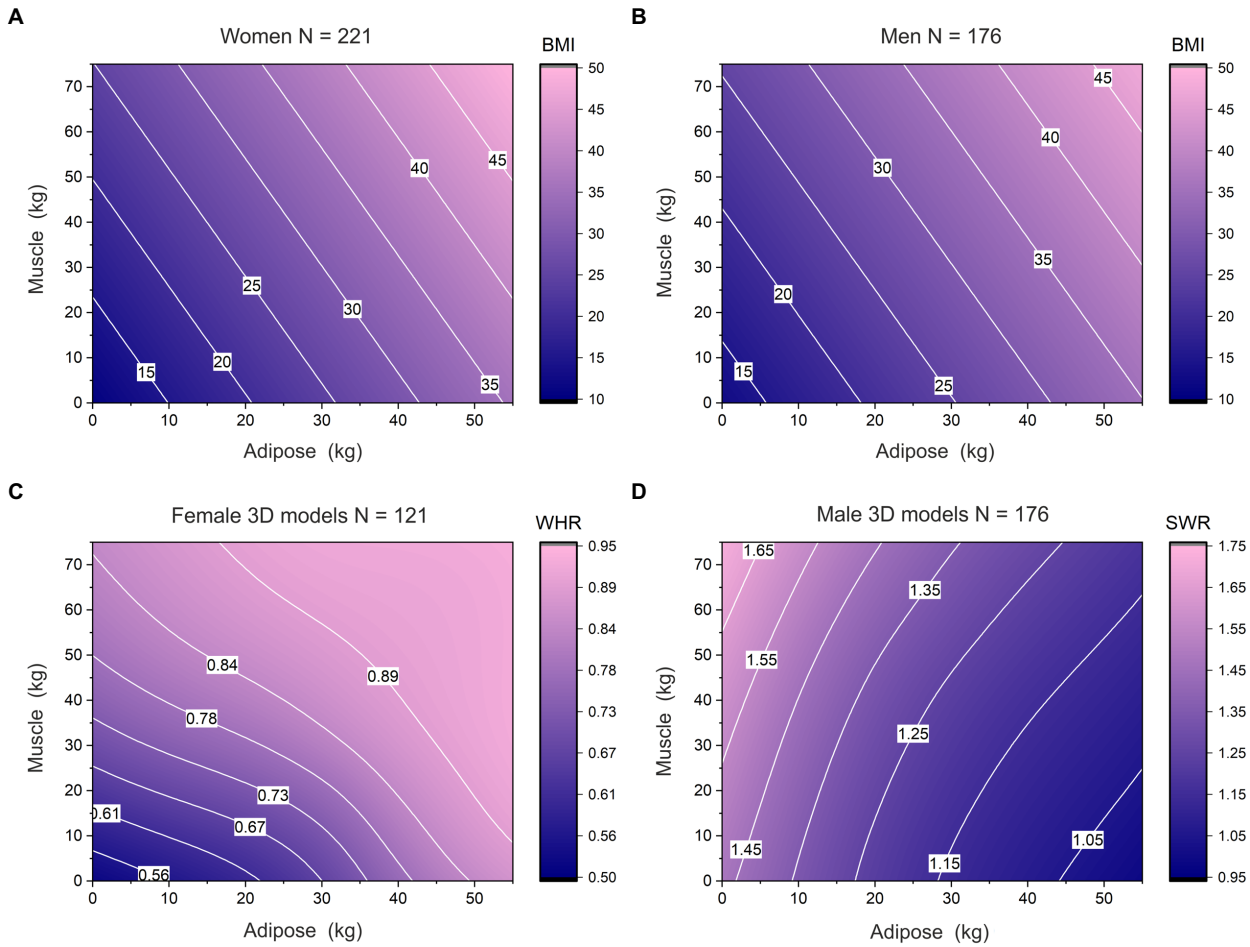
Contour plots showing the relationships between body composition (i.e., muscle mass on the *y*-axis and adipose mass on the *x*-axis) and BMI (a and b), WHR **(C)**, and SWR **(D)**. In each case, BMI increases from navy blue to pink. The isocontours, in white, show how varying combinations of muscle and adipose mass can give rise to the same BMI, WHR, or SWR values. The plots in the top row are derived from multiple regression models predicting BMI from muscle mass, adipose mass, and chronological age in 221 women **(A)** and 176 men **(B)** (see, Maalin et al., 2021). The plots in the bottom row are derived from direct measurement of the 3D stimuli used in this study: WHR for 121 female stimuli (c) and SWR for 121 male stimuli (d). In each case for (c) and (d) we calculated smoothed spline interpolations (Harder and Desmarais, 1972; Meinguet, 1979; Green and Silverman, 1994) using PROC MIXED in SAS v9.4 (SAS Institute, North Carolina, United States).

Studies of physical attractiveness have tended to use body stimuli which systematically vary in indices of body shape and use these indices to predict attractiveness judgements. Such indices include: the waist-to-hip ratio [Waist-to Hip Ratio (WHR); (Singh, 1993; Lassek and Gaulin, 2016; Bovet, 2019)], shoulder-to-waist ratio [Shoulder-to-Waist Ratio (SWR); (Anderson et al., 2004; Andrews et al., 2017; Burch and Johnsen, 2020)], and body size/mass, indexed by BMI as a proxy for body fat (Tovée et al., 2002, 2016; Smith et al., 2007). However, all of these measures are problematic. First, BMI is not an accurate index of body fat [see (Gardner and Brown, 2010; Groves et al., 2019)]. The problem here is that differences in BMI across individuals are driven by a combination of *both* body fat and skeletal muscle mass, i.e., body composition (Sturman et al., 2017). Indeed, the relationship between body composition and BMI represents a clear example of Simpson’s paradox (Simpson, 1951). This is illustrated by 2D scatterplots of muscle mass as a function of body fat. In any reasonably large sample of men or women, this relationship shows a positive correlation across the sample. However, if the data are subdivided into narrow ranges of BMI, then the direction of the relationship between muscle and adipose mass, within each narrow BMI band, inverts and becomes negatively correlated. This phenomenon is illustrated by the white contours in Figure 1A (women) and 1b (men). They illustrate how different individuals can have the same BMI but different body composition (i.e., higher muscle mass with lower body fat, or vice versa), and therefore different body shapes (Yajnik and Yudkin, 2004; Mullie et al., 2008). The same problem exists for other indices of body shape. Figures 1C,D illustrate an equivalent problem for two commonly used body shape metrics: WHR in women (Figure 1C) and SWR in men (Figure 1D). A number of different body compositions can give rise to the same body shape metric. So, although previous studies have assumed that a particular body shape signals a particular composition (Singh, 1993; Maisey et al., 1999; Fan et al., 2005; Hönekopp et al., 2007; Gardner and Brown, 2010; Sell et al., 2017), this is likely a false assumption. Consequently, in the present study, we vary the body composition of body stimuli based on data from real men and women to avoid such ambiguity.

To do this, we generated 121 Computer Generated Imagery (CGI) male and 121 CGI female bodies using the method of Maalin et al. (2021). This allows participants to make attractiveness judgements about a set of bodies that completely tile the 2D body composition space, with one dimension corresponding to increasing adipose mass, and the second orthogonal dimension corresponding to increasing muscle mass.

To quantify the degree to which sociocultural pressures are internalized by our participants, we used the Sociocultural Attitudes Towards Appearance Questionnaire (SATAQ-4; Schaefer et al., 2015) which indexes internalization of muscularity and thinness ideals, and media, peer and family pressures. We added some additional measures including the Eating Disorder Examination Questionnaire (EDE-Q; Fairburn and Beglin, 1994) whose subscales allow us to quantify a range of features including weight and body shape concerns, as well as the Beck Depression Inventory as a measure of depressive symptomatology (BDI; Beck et al., 1961). The reason for including such additional measures is because recent research has shown that when individuals make judgements of other women’s body sizes, for example, then their judgements are partially influenced by their own body concerns, and we wanted to control for such effects statistically (Cornelissen et al., 2022). For similar reasons, we also included the Drive for Muscularity Scale (DMS; McCreary and Sasse, 2000) as a further measure of the raters’ own muscularity concerns. This set of psychometric measures therefore provides a good catalog of the pressures and concerns that may predict body preferences from the point of view of the rater absorbing information from the environment, as well as influences derived from their own body image status.

We carried out two studies. In the first, male and female participants rated all 242 male and female bodies varying in muscle and adipose for attractiveness. This allowed us to characterize changes in attractiveness across the complete body composition space. This is important, because it allows us to distinguish between a number of possibilities including: (a) a single peak for attractiveness or more than one peak in body composition space; (b) threshold or shelf effects whereby above certain adipose/muscle mass combinations, all images are treated as equivalently attractive; and (c) ridge effects whereby certain systematic combinations of adipose and muscle, e.g., changing from higher adipose/lower muscle through to lower adipose/higher muscle, might be treated as equivalently attractive (akin to the isocontours in Figure 1). In the second study, we asked our participants to choose the most attractive male and female bodies in a Method of Adjustment (MoA) task in which they could independently vary muscle and adipose to create the most attractive body. This allowed us to: (a) compare these locations for peak attractiveness in body composition space to published norms for what constitutes the median body composition for men and women’s actual bodies; and (b) investigate the degree to which individual differences in internalization of thinness and muscularity predict how muscled and how thin the most attractive bodies are. The results of these studies allow us to test whether individual differences in internalization of cultural ideals, drive for muscularity, eating disorder symptomatology and depressive symptoms systematically shift the location of peak attractiveness in body composition space. We hypothesize that the degree to which cultural ideals are internalized will be the primary driver of a preference for high muscle and low fat bodies for both genders.

## Study 1: Judgements of attractiveness of all bodies

### Methods

#### Ethics statement

The Department of Psychology ethical committee at Northumbria University and the Business, Law and Social Sciences Faculty Academic Ethics Committee at Birmingham City University granted ethical approval for this study.

#### Sample size

To date, a number of studies have examined attractiveness and body preference based on attributes including body composition (Cafri et al., 2004; Yanover and Thompson, 2010; Brierley et al., 2016; Sell et al., 2017; Sturman et al., 2017; Lei et al., 2019; Talbot et al., 2019; Voges et al., 2019; Lei and Perrett, 2021), with sample sizes of raters varying from 9 to 2,733 per gender group (median = 58.5). None of these studies has attempted power calculations to estimate sample size. Moreover, in the current study, we intended to use multivariate techniques such as linear mixed effects modelling and multivariate multiple regression. To our knowledge, there is no precedent set to estimate statistical power using such techniques for multivariate outcomes such as body composition. While it is true that simulation techniques are available [see, e.g., (Kumle et al., 2021)], reliable simulations need reasonable estimates of the various sources of variance and covariance which we do not have. Therefore, our approach here is not to rely *solely* on inferential statistical methods that estimate probability, and which require adequate power. Instead, we will focus primarily on model building through testing changes in the Akaike information criterion (AIC; Akaike, 1973) to compare the adequacy of multiple models as a fit to our data (Burnham and Anderson, 2002; Wagenmakers and Farrell, 2004). Final sample size was determined by the time allocated to the first author to collect the data (Lakens, 2022).

#### Participants

Opportunity sampling was used to recruit 69 White females (chronological age *M* = 26.13 years, SD = 9.53) and 69 White males (chronological age *M* = 38.30 years, SD = 13.28) for the study. Both Northumbria University and Birmingham City University’s SONA system was used for recruitment, as well as advertising the study on social media. Prolific was used to recruit most of the male subjects, which may explain why the age of male subjects is higher than that of females. Of the male participants, 58 were recruited *via* Prolific (chronological age *M* = 39.45 years, SD = 13.27) and 11 were recruited using the other strategies (chronological age *M* = 32.27 years, SD = 12.18). It is however important to note that age was included as a covariate when modelling participants’ responses. Participants were eligible to take part if they were over 18, understood written English and were not currently, or previously, diagnosed with an eating disorder. The sample of Caucasian participants was not restricted to a typical student population and covered a wide age range from 19 to 71.

#### Stimuli and attractiveness rating task

**Figure 2:**
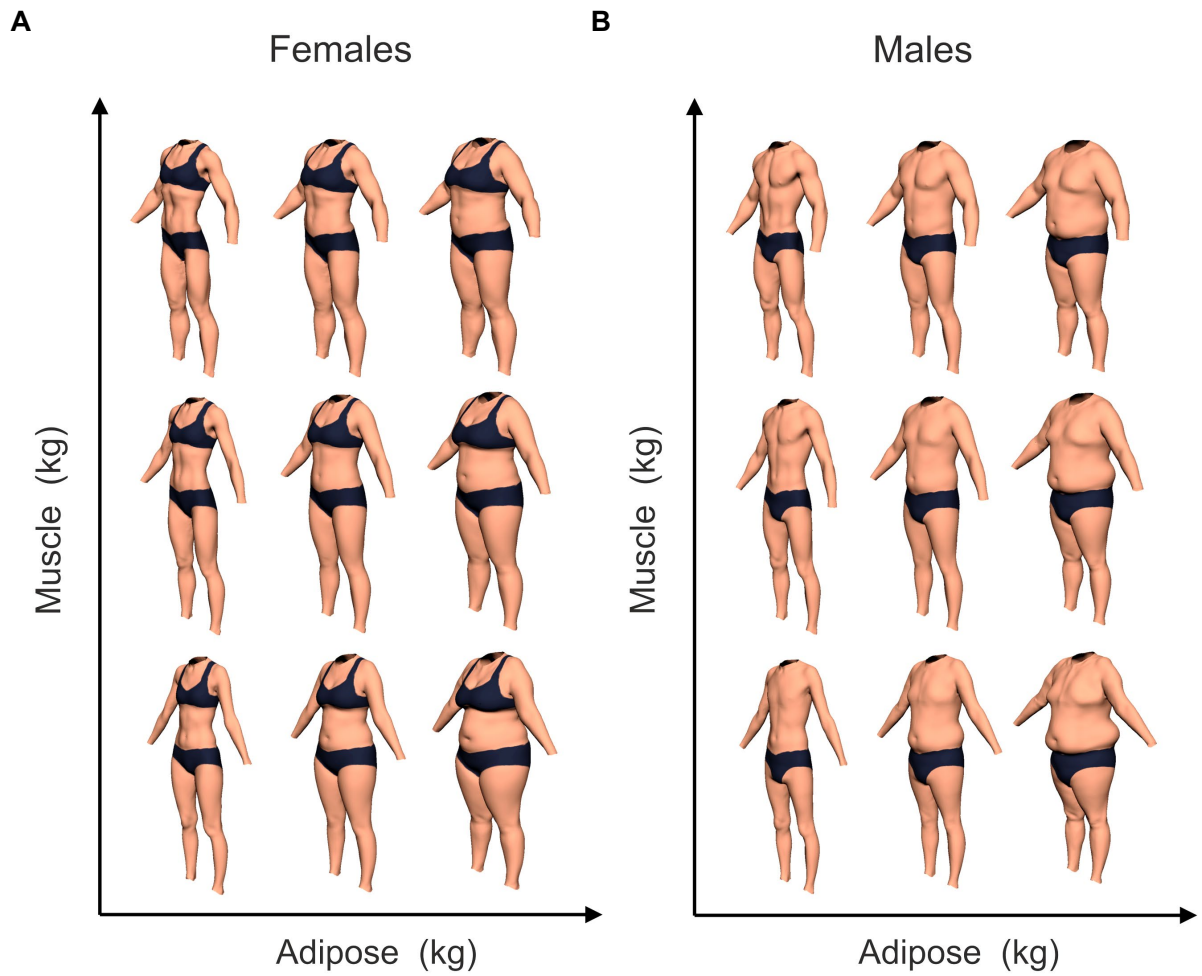
Examples of the CGI bodies used in this study to illustrate the changes of body shape and size of the female stimuli **(A)** and male stimuli **(B)** produced by changing their body composition based on our PCA of the 3D scanned bodies. The bodies in each set are grouped into three columns, from left to right: low, mid, and high muscle tone. They are further divided into three rows from bottom to top: low, mid, and high muscle mass.

All participants were asked to rate the attractiveness of 3D computer generated bodies of which there were 121 females and 121 males. Each body was presented in an image showing three viewpoints (front, three-quarter view and profile), to make sure all the visual cues to both muscle and adipose content were visible to the observer. Examples of the stimuli in the three-quarter view are shown in Figure 2, which illustrates how body composition changes body size and shape. Participants rated all 121 females and all 121 male bodies in separate blocks, and the order of presentation of the blocks was randomized across participants. In addition, the order of images within a block was also randomized for each participant. The sets of stimuli were created using the techniques outlined by Maalin et al. (2021), which produces bodies biometrically calibrated for body composition. To achieve this, Maalin et al. (2021) obtained high-resolution 3D body shape scans and bioimpedance body composition measures from 221 women and 176 men, and produced a statistical mapping between the two, separately for men and women. This allows the creation of 3D computer-generated models of bodies that substantially improve the accuracy and precision with which assessments of body size and shape can be made. The bodies used in this study represent systematic additions of adipose and muscle to a minimal baseline body shape. All possible combinations of 0–55 kg increments of adipose, in 5.5 kg steps, were made together with 0–75 kg increments of muscle, in 7.5 kg steps. Each of the 242 images was presented individually to the participant and they were asked to use a Likert scale to rate the attractiveness of the bodies from extremely unattractive (1) to extremely attractive (10). The attractiveness judgement is not otherwise defined.

#### Psychometric measures

A set of four psychological measures was introduced as a “buffer” between the tasks in this study so that the participants did not have to rate all 240 images in one go. The order of presentation of the psychometric measures were also randomized.

Sociocultural Attitudes Towards Appearance Questionnaire-4 (SATAQ-4; Schaefer et al., 2015):

The SATAQ-4 is a 22-item measure that assesses internalization of appearance ideals and appearance related pressures. Attitudes to one’s appearance are measured in the questionnaire by five sub-scales: two for internalization of ideals (thin/low body fat and athletic/muscular dimensions) and three for pressures (media, peers, and family dimensions). Items are rated using a five-point Likert scale with response options ranging from 1 (definitely disagree) to 5 (definitely agree). Higher scores on these measures indicate greater internalization and acceptance of societal appearance ideals, as well as increasing pressures from family, peers, and media. In the current sample, Cronbach’s alpha for SATAQ-4 was 0.89.

##### Eating disorder examination questionnaire (EDE-Q)

The EDE-Q is a self-report questionnaire containing four subscales: (1) the Restraint (EDE-Q res) subscale containing 5 items which measure the restrictive nature of eating; (2) the Eating Concern (EDE-Q eat) subscale including 5 items measuring preoccupation with food and social eating; (3) the Shape Concern (EDE-Q sc) subscale containing 8 items which measure body shape dissatisfaction; and (4) the Weight Concern (EDE-Q wc) subscale containing 5 items measuring dissatisfaction with body weight. Participants must report how many days within the past 4 weeks (28 days) they have been concerned by each of the items, e.g., Have you had a definite desire to have an empty stomach with the aim of influencing your shape or weight? The 7-point response scale ranges from 0 indicating no days to 6 indicating every day. There are also six frequency items that require participants to state themselves how many times or how many days they have experienced each item, e.g., Over the past 28 days, how many times have you made yourself sick (vomit) as a means of controlling your shape or weight? Frequency data on the key behavioral features are noted and a global score of the overall disordered eating behavior is calculated by averaging the appropriate items, with a higher score indicating higher disordered eating pathology. The internal consistency of the EDE-Q in this current sample was 0.94 (Fairburn and Beglin, 1994).

##### Drive for muscularity scale

The DMS is a 15-item self-report measure that assesses an individual’s concerns, attitudes and behaviors in response to the societal pressure to achieve a mesomorphic body shape (i.e., muscular, and lean). Participants are asked to indicate the extent to which they feel a series of attitudes and behaviors are descriptive of themselves, for example, I feel guilty if I miss a weight session. A six-point response scale is used to rate each item, ranging from 1 (never) to 6 (always), with higher scores indicating higher levels of drive for muscularity. The DMS has shown consistently acceptable reliability in both male and female respondents, with alpha reliability estimates ranging from 0.85 to 0.91 in males and above 0.80 in females (McCreary, 2007). Internal consistency for this measure in the current sample was 0.94 (McCreary and Sasse, 2000).

##### Beck depression inventory

The BDI consists of 21 items to assess the intensity of depressive symptomatology. The measure includes items relating to symptoms of depression such as irritability, cognitions such as guilt, and physical symptoms such as fatigue and lack of interest in sex. Each item has a set of four possible options, ranging in intensity from 0 to 3. A total score is created and compared to a key to determine the depression severity, with the standard cut off scores being as follows: 0 to 9, minimal depression; 10 to 18, mild depression; 19 to 29, moderate depression and 30 to 63, severe depression. Cronbach’s alpha for the BDI in the current sample was 0.92 (Beck et al., 1961).

#### Procedure

Participants clicked on a link to Qualtrics and were presented a description of the study, which gave them enough information to consent to take part. After this, participants were requested to self-report their age, gender, ethnicity, weight (in stones and pounds, kilograms, or pounds) and height (in feet and inches, or meters, or centimeters). Confirmation that they did not have a current or previous diagnosis of an eating disorder was also obtained to ensure that those who might experience psychological discomfort during the study were excluded. They then were asked to rate the first set of 121 bodies for attractiveness, complete the four psychometric questionnaires, and then rate the second set of 121 bodies for attractiveness. Participants were randomly assigned to rate either the male or the female bodies for the first rating block. They then rated images of the other sex for the second rating block. Once they completed the tasks, participants were presented with the study debrief. This entire procedure took approximately 30 min to complete.

## Results

### Univariate statistics

Participant characteristics and scores on the psychometric measures for Study 1 are summarized in Table 1.

**Table 1:** Participant characteristics and psychometric scores for Study 1.

	Females *n* = 69		Males *n* = 69	
	Mean	SD	Mean	SD
BMI	26.41	16.37	27.43	5.34
DMS	29.57	12.46	32.19	16.67
BDI	13.59	10.78	11.39	10.05
EDE-Q Global	2.17	1.37	1.27	1.04
EDE-Q Eating Concern	1.35	1.33	0.46	0.76
EDE-Q Restraint	1.54	1.52	1.19	1.52
EDE-Q Shape concern	3.07	1.66	1.87	1.51
EDE-Q Weight concern	2.72	1.79	1.58	1.32
SATAQ Thinness	15.78	5.44	11.52	4.87
SATAQ Muscular	13.30	5.42	12.04	5.93
SATAQ Media pressure	16.23	3.39	11.00	6.04
SATAQ Peer pressure	8.10	4.56	6.80	4.05
SATAQ Family pressure	25.45	26.11	24.13	26.17

### Multivariate statistics; judgements of images of females

**Figure 3:**
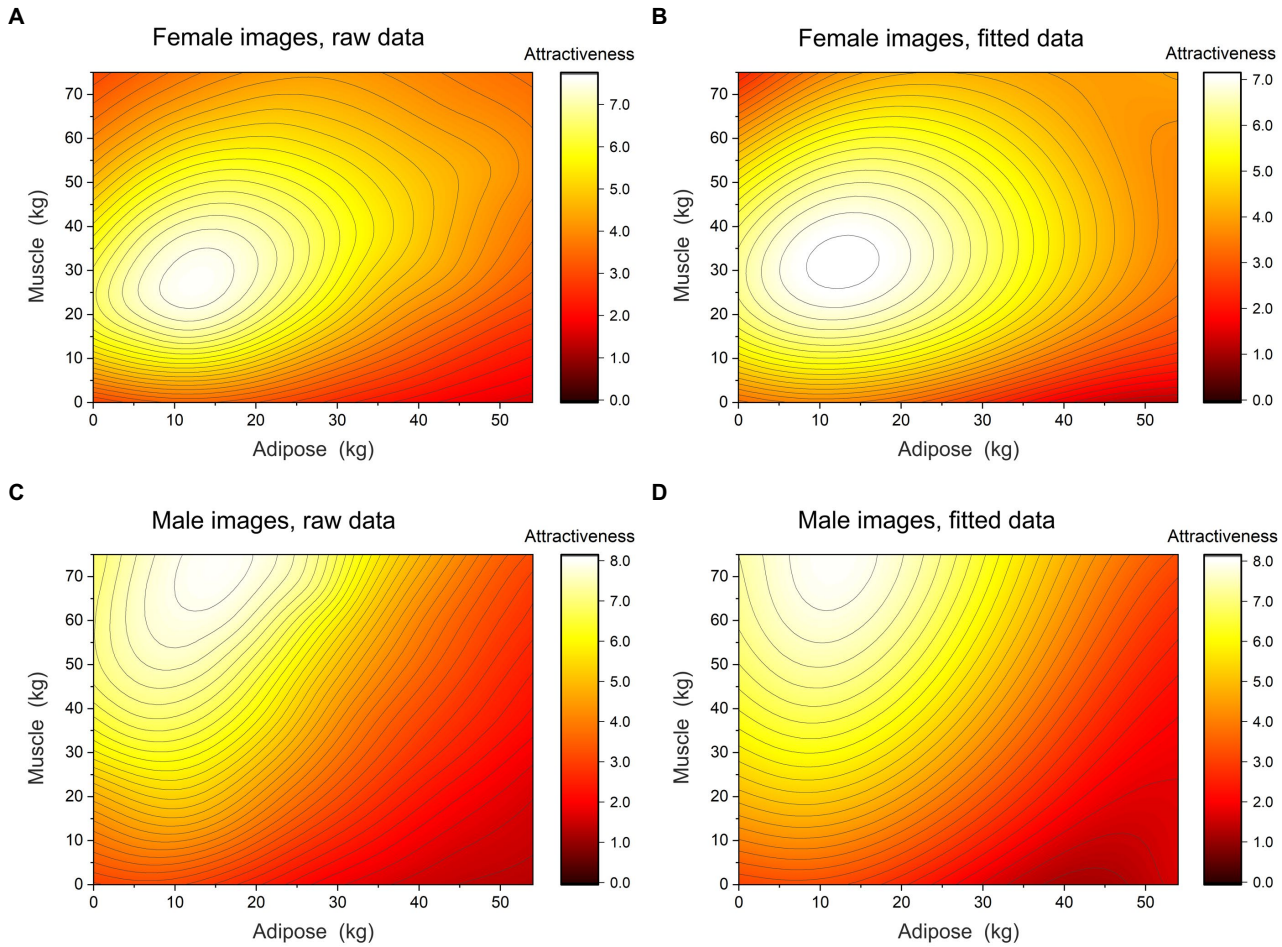
The left column shows contour plots of attractiveness as a function of muscle and adipose mass, averaged across male and female observers, separately for **(A)**, female and **(C)**, male images. The right column shows contour plots of model fitted attractiveness as a function of muscle and adipose mass, averaged across male and female observes, separately for **(B)**, female and **(D)**, male images. In each plot, attractiveness increases systematically from brown through red, and yellow to white.

We wanted to estimate statistical models that capture the relationship between the attractiveness ratings of female bodies, their body composition, the age, sex, and BMI of the raters, and the raters’ psychometric performance. Figure 3A shows a contour plot of attractiveness ratings, averaged across raters, as a function of the skeletal muscle and adipose mass of the female stimuli. It is clear from this plot of the raw data that polynomial terms for both fat and muscle are needed to model these non-linear relationships.

To do this, we built a succession of linear mixed effects models of attractiveness ratings, using PROC MIXED in SAS v9.4 (SAS Institute, North Carolina, USA). First, we fitted an empty model with only random effects for the intercept and for the slope terms for adipose and muscle at the participant level. Next, we added one explanatory variable at a time, checking whether the addition of each new variable reduced AICc from one model to the next [AICc is preferable to AIC for smaller samples (Hurvich and Tsai, 1989)]. The larger the Δ AICc*_i_*, the less plausible is the fitted model *i* as being the next best approximating model in a candidate set. To decide whether to retain a new explanatory variable, we adopted the criteria of Burnham and Anderson (2002): model comparisons where Δ AICc*_i_* ≤ 2 have no support (evidence) for retaining a new variable, those in which 4 ≤ Δ AICc*_i_* ≤ 7 have considerable support, and models having Δ AICc*_i_* > 10 have substantial support. For judgements of female bodies, we added linear, then quadratic, then cubic polynomial terms for the fixed effects of muscle and adipose mass, and then explored interactions between the two. Note that to avoid scaling problems for the estimation of very small regression weights, the values for adipose and muscle mass were divided by 10. Once the iterative model fitting process was complete for adipose and muscle mass, we tested the viability of additional fixed effects for: rater sex, rater age, and BMI of the rater, as well as rater performance on the DMS, EDE-Q, BDI, and SATAQ psychometric measures. This process is summarized in Supplementary Table S1 of the Supplementary materials. The final model, which contains confidence intervals and probability values, is reported in Table 2. Note that no fixed effects beyond those for adipose and muscle mass were retained. The model is illustrated by the contour plot of fitted attractiveness in Figure 3B.

**Table 2:** Linear mixed effects model of responses to images of females.

Model effects	*t* value (*df*)	*Z* value	*p* value	Parameter estimate	95% CI
Random effects					
Intercept	23.90 (206)		<0.0001	3.05	2.79–3.30
Adipose	15.37 (2320)		<0.0001	1.08	0.94–1.22
Muscle	54.23 (4895)		<0.0001	2.46	2.37–2.55
Adipose^2^	−28.07 (16000)		<0.0001	−0.65	−0.69––0.60
Muscle^2^	−43.78 (16000)		<0.0001	−0.53	−0.55––0.51
Adipose^3^	26.58 (16000)		<0.0001	0.070	0.065–0.075
Muscle^3^	23.43 (16000)		<0.0001	0.024	0.022–0.026
Adipose × Muscle	16.90 (16000)		<0.0001	0.16	0.14–0.18
Adipose^2^ × Muscle	−25.47 (16000)		<0.0001	−0.058	−0.063––0.054
Adipose^2^ × Muscle^2^	30.12 (16000)		<0.0001	0.006	0.006–0.007
Random effects					
Ppt variance (intercept)		7.90	<0.0001	1.74	
Ppt Adipose covariance		−6.01	<0.0001	−0.33	
Adipose variance (slope)		8.04	<0.0001	0.15	
Muscle Ppt covariance		−0.74	0.5	−0.018	
Muscle Adipose covariance		−3.69	0.0002	−0.027	
Muscle variance (slope)		7.76	<0.0001	0.039	

Ppt, participant.

Variance explained for a linear mixed effects model is more complex compared with a single level model, since there are multiple residual terms (Lorah, 2018). A formula for *R*^2^ specific to such models is provided by Snijders and Bosker (2012). It represents proportional reduction in prediction error at the individual level:


$$R^{2}=1-\frac{\sigma_{F}^{2}+\tau_{F}^{2}}{\sigma_{E}^{2}+\tau_{E}^{2}},$$

where $\sigma_{F}^{2}$ corresponds to the level-one random error variance (variance of *e_ij_*) for the full model (i.e., the model of interest); $\tau_{F}^{2}$ corresponds to the level-two random error variance (variance of *u_0j_*) for the full model; $\sigma_{E}^{2}$ corresponds to the level-one random error variance for the empty model; and $\tau_{E}^{2}$ corresponds to the level-two random error variance for the empty model. Accordingly, the model in Table 2 for images of females, explains 37% of the variance in attractiveness ratings relative to the unexplained variance in attractiveness ratings. Furthermore, the effect size measure related to variance explained for the overall model is *f* ^2^ which can be computed as (Cohen, 1992):


$$f^{2}=\frac{R^{2}}{1-R^{2}}.$$

In the present study, *f* ^2^ = 0.59 for the overall model for images of females in Experiment 1. Guidelines for interpretation of *f*  ^2^ indicate that 0.02 is a small effect, 0.15 is a medium effect, and 0.35 is a large effect (Cohen, 1992), indicating that the present effect is large.

### Multivariate statistics; judgements of images of males

Figure 3C shows a contour plot of attractiveness ratings, averaged across raters, as a function of the skeletal muscle and adipose mass of the male stimuli. Again, this illustrates the need for polynomial terms in the model for both skeletal muscle and adipose mass. We followed the same modelling procedure as for the female images, and the iterative process of model building and selection through changes in AICc is summarized in Supplementary Table S2 of the Supplementary materials. Table 3 shows the parameter estimates for the fixed and random effects of the final linear mixed effects model. Note that no fixed effects beyond those for adipose and muscle mass were retained. The final model is illustrated by the contour plot of fitted attractiveness in Figure 3D. The overall model for images of males explained 62.6% of the variance in attractiveness ratings relative to the unexplained variance in attractiveness ratings. The effect size, *f^2^* = 1.67, corresponds to a large effect size.

**Table 3:** Linear mixed effects model of responses to images of males.

Model effects	*t* value (*df*)	*Z* value	*p* value	Parameter estimate	95% CI
Fixed effects					
Intercept	24.6 (184)		<0.0001	2.97	2.73–3.21
Adipose	12.7 (1826)		<0.0001	0.77	0.65–0.89
Muscle	27.0 (3423)		<0.0001	1.07	0.99–1.15
Adipose^2^	−34.0 (16000)		<0.0001	−0.67	−0.71––0.63
Muscle^2^	−4.76 (16000)		<0.0001	−0.049	−0.069––0.029
Adipose^3^	39.8 (16000)		<0.0001	0.090	0.085–0.094
Muscle^3^	−2.80 (16000)		0.005	−0.0025	−0.0042––0.00075
Adipose × muscle	16.7 (16000)		<0.0001	0.13	0.12–0.15
Adipose^2^ × muscle	−34.0 (16000)		<0.0001	−0.066	−0.070––0.062
Adipose^2^ × muscle^2^	21.5 (16000)		<0.0001	0.0039	0.0035–0.0042
Random effects					
Ppt variance (intercept)		7.87	<0.0001	1.60	
Ppt adipose covariance		−5.96	<0.0001	−0.28	
Adipose variance (slope)		7.97	<0.0001	0.13	
Muscle Ppt covariance		−2.44	0.01	−0.055	
Muscle adipose covariance		−3.65	0.0003	−0.024	
Muscle variance (slope)		7.78	<0.0001	0.036	

Ppt, participant.

## Discussion of study 1

The averaged attractiveness ratings for female images in Figure 3A, and the fitted data in Figure 3B, suggest that the women rated most attractive inhabit a broad maximum in body composition space, like a hilltop. In all directions from this peak, for 360°, attractiveness smoothly declines. There is no compelling evidence for secondary peaks, shelves or ridges in this space. The statistical modelling reported in Supplementary Table S1 makes it clear that no fixed effects beyond those relating to body fat and muscle mass contribute to the structure of the fitted surface. The equivalent averages and fitted data for images of men in Figures 3C,D suggest a similarly shaped peak at higher muscularity than for images of women, but similar adiposity. Again, there is little evidence for secondary peaks, shelves or ridges. However, it does appear that there may be a ceiling effect for attractiveness at the highest muscularity. Since there were no stimuli with skeletal muscle increments above 75 kg, it is therefore unclear whether the highest attractiveness ratings for images of men would persist for even more muscular images, or whether attractiveness would start to decline. The statistical modelling reported in Supplementary Table S2 shows that, as with the images of females, no fixed effects beyond those relating to body fat and muscle mass contribute to the structure of the fitted surface.

At face value, Study 1 suggests that there are no obvious influences of raters’ psychometric performance on their attractiveness judgements. However, it is worth bearing in mind what a statistically significant main effect of SATAQ media or raters’ BMI, for example, would mean here. Essentially, a main effect of SATAQ media would cause the whole fitted surface to “rise” systematically, but not change its overall shape, with increasing SATAQ scores. Even an interaction between adipose mass and SATAQ media would merely tilt the whole surface. What we would really like to know is whether increasing internalization of media information, for example, leads women to rate men who have less body fat and more muscle, as most attractive? In other words, we need to test whether individual differences in SATAQ media scores, for example, drive systematic shifts in the location of peak attractiveness in body composition space. This, in turn, would amount to a systematic shape change of the 3D surfaces described in Figure 3 across individuals, rather than a translation or tilt applied to the whole surface. Therefore, to do this in Study 2, we used the same stimulus set as in Study 1, but this time incorporated the stimuli into a method of adjustment (MoA) task, in which participants could manipulate muscularity and adiposity independently, in the search for the most attractive male and female image.

## Study 2: Judgments of attractiveness using the method of adjustment task

### Methods

#### Participants

The same recruitment strategy and exclusion criteria were used as in Study 1. For Study 2, of those who fully completed the study, 65 identified themselves as White female (chronological age *M* = 22.57 years, SD = 5.47) and 65 as White male (chronological age *M* = 31.63 years, SD = 11.83). Participants ranged from age 19 to 56, therefore not restricting the sample to a typical student population. As in study 1, Prolific was also used to support the recruitment of additional male subjects with 34 males recruited from Prolific (chronological age *M* = 37.68 years, SD = 11.33) and 31 recruited *via* social media and SONA (chronological age *M* = 25.00 years, SD = 8.39). The use of Prolific can again explain why the average age of male subjects is higher than that of females. Although the mean age of males was higher, age was controlled for in the modelling of participants’ responses.

#### Stimuli and method of adjustment task

The same set of stimuli were used in Study 2, as in Study 1, which were created using the method of Maalin et al. (2021). In Study 2, however, they were presented as a MoA task. On each trial of the MoA, participants were required to find which body within the 2D continuum of muscle and adipose they believed to be the most attractive male and female bodies. To search this space, participants were presented with a single image on screen, and they could press the left/right and up/down arrow keys. A single left/right arrow key press reduced/increased body fat by 5.5 kg to a maximum of 55 kg. A single up/down arrow key press increased/reduced muscle mass by 7.5 kg to a maximum of 75 kg. At the start of each trial, the body composition of the avatar was set to a random location in the 2D body composition space. Participants carried out two blocks of ten trials (five for each stimulus sex), separated by the psychometric measurements. In each block of trials, the sex of the presented image alternated systematically between male and female from one trial to the next.

#### Psychometric measures

The same four psychometric measures were used as in Study 1 as a buffer between the repeats of the MoA task: SATAQ-4, EDE-Q, DMS and BDI. The Cronbach’s alpha values for the psychometric measures were: 0.91, 0.95, 0.90, and 0.93, respectively.

#### Procedure

Once participants had clicked on the link to Qualtrics, they were presented with a description of the study, which gave them enough information to consent to take part. At this point, participants were asked to confirm that they were using either a laptop or desktop PC to complete the study, rather than a mobile phone or tablet. If participants declared that they were trying to use an ineligible device, they were directed to the end of the survey where it was explained that they did not meet the eligibility criteria to continue. If a participant claimed that they were using an eligible device but were actually using a mobile phone or tablet, the software running on Pavlovia.org detected and recorded this and these participants’ data were excluded from the study. After this, participants were requested to self-report their age, gender, ethnicity, weight (in stones and pounds, kilograms, or pounds) and height (in feet and inches, or meters, or centimeters). Confirmation that they did not have a current or previous diagnosis of an eating disorder was also obtained to ensure that those who might experience psychological discomfort during the study were excluded. Participants were then directed to Pavlovia.org where they were asked to carry out the MoA task to choose the body within the 2D continuum that they judged as most attractive. After repeating this task five times for each image sex, participants were directed back to Qualtrics where they were required to complete the four psychometric measures. The psychometric measures acted as a buffer, as they were then re-directed back to Pavlovia.org a further time to repeat the MoA task, again five times for each image sex. Then, participants were redirected to Qualtrics to be debriefed and the study ended. This entire procedure took approximately 30 min to complete.

## Results

### Univariate statistics

Participant characteristics and scores for psychometric measures for Study 2 are summarized in Table 4.

**Table 4:** Participant characteristics and psychometric scores for Study 2.

	Females *n* = 65		Males *n* = 65	
	Mean	SD	Mean	SD
BMI	22.27	3.68	24.86	4.93
DMS	29.54	10.22	40.74	12.83
BDI	11.85	10.34	13.49	10.50
EDE-Q Global	1.71	1.33	1.36	1.23
EDE-Q eating concern	1.24	1.36	1.09	1.44
EDE-Q restraint	0.96	1.18	0.59	0.94
EDE-Q shape concern	2.49	1.63	2.02	1.64
EDE-Q weight concern	2.14	1.57	1.75	1.49
SATAQ thinness	16.38	5.01	13.29	4.39
SATAQ muscular	12.51	4.82	14.29	5.47
SATAQ media pressure	15.00	4.48	10.75	4.65
SATAQ peer pressure	7.94	4.24	7.37	3.58
SATAQ family pressure	31.05	26.77	26.74	25.78

### Multivariate statistics

**Figure 4:**
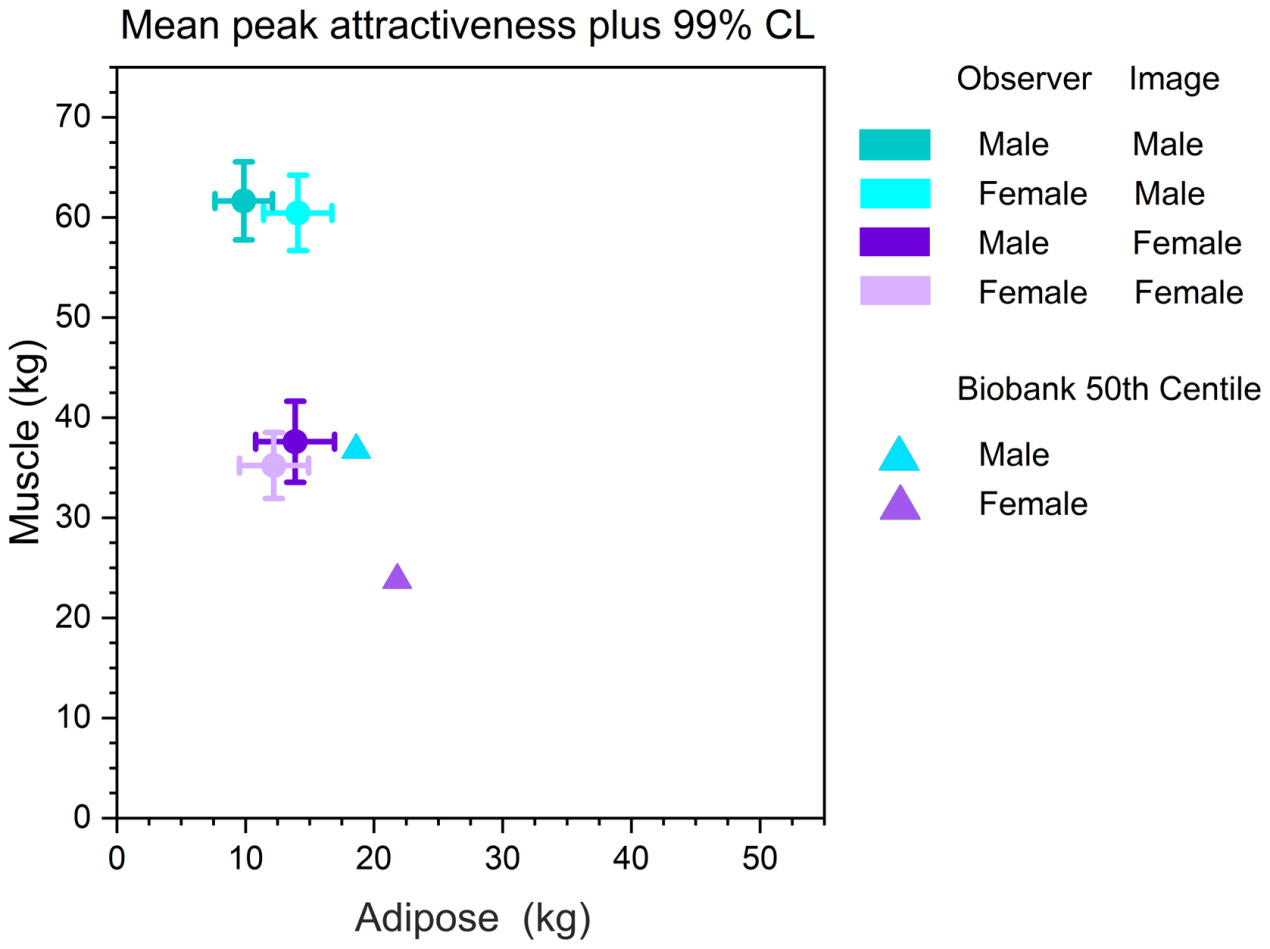
Circles show locations in body composition space for the most attractive bodies, shown separately for images of male and female bodies, and male and female observers. The error bars correspond to 99% confidence intervals for both body fat and skeletal muscle mass. The triangles represent the 50th centiles of total skeletal muscle mass and body fat for adult White males and females in the UK Biobank.

Figure 4 shows a plot of the mean peak attractiveness locations in body composition space, shown separately for male and female raters as well as for male and female stimulus images. As a reference, we also plot the 50th centile values for total skeletal muscle mass and body fat for men and women, derived from 375,512 White participants in the UK Biobank (Lee et al., 2020). To do this, we first used a factor of 0.75 (Gallagher et al., 1997) to convert the appendicular skeletal muscle mass indices reported in Lee et al. (2020) to total muscle mass indices, and then used the average heights for United Kingdom men and women (Health Survey for England, 2012) to convert muscle and fat indices into equivalent kilogram masses. Consistent with the peaks in Figures 3A,C, it is clear from Figure 4 that, on average, the most attractive male bodies had substantially more muscle mass, and marginally less body fat than the most attractive female bodies. Moreover, it is very clear that on average, both male and female observers found bodies with less adipose and substantially more muscle than the UK population norms, as most attractive.

The MoA task we used resulted in a multivariate dataset with four outcome measures (i.e., female image adipose, female image muscle, male image adipose, and male image muscle, all at peak attractiveness) together with a number of explanatory variables including: rater sex, age, and BMI, as well as raters’ psychometric performance (DMS, BDI, EDE-Q, and SATAQ). Unfortunately, a technical error on Pavlovia.org meant that the second block of MoA trials was not saved to file, therefore our analysis is based on the first block of trials only. Owing to the repeated measures multivariate design, where each rater judged images of both men and women, we used PROC MIXED in SAS v9.4 (SAS Institute, North Carolina, United States) to compute multivariate multiple regression models. To do this, we generated a class variable (named Var in this paper) to identify the four levels of the outcome measure. The Var variable generates four design matrix columns corresponding to four intercept terms, one for each outcome. Therefore, we also used the NOINT option in the MODEL statement to prevent PROC MIXED from generating another, unnecessary intercept column. In general, Var is crossed with each other effect in the model. For model optimization, as with Study 1, we started with an empty model, and added/removed explanatory variables depending on whether the addition of a new variable substantially reduced AICc or not (see Supplementary Table S3; Supplementary materials). Table 5 shows the results of the full multivariate multiple regression model, selected on this basis. It explained 63.7% of the variance in attractiveness ratings relative to the unexplained variance in attractiveness ratings, with an *f* ^2^ = 1.75, corresponding to a large effect size.

**Table 5:** Multivariate multiple regression.

Model effects	Var	Rater sex	Estimate	SE	*t* value (*df*)	*p* value
Fixed effects						
Var	Adip_F-image		−1.04	4.88	−0.21 (130)	0.8
Var	Adip_M-image		3.33	4.60	0.72 (130)	0.5
Var	Musc_F-image		19.25	6.88	2.80 (130)	0.006
Var	Musc_M-image		34.08	6.90	4.94 (130)	<0.0001
Var × Rater sex	Adip_F-image	F	1.31	1.65	0.80 (130)	0.4
Var × Rater sex	Adip_F-image	M	0.00	.	.	.
Var × Rater sex	Adip_M-image	F	5.66	1.55	3.65 (130)	0.0004
Var × Rater sex	Adip_M-image	M	0.00	.	.	.
Var × Rater sex	Musc_F-image	F	1.72	2.32	0.74 (130)	0.5
Var × Rater sex	Musc_F-image	M	0.00	.	.	.
Var × Rater sex	Musc_M-image	F	4.13	2.33	1.77 (130)	0.08
Var × Rater sex	Musc_M-image	M	0.00	.	.	.
Var × Age	Adip_F-image		0.083	0.079	1.04 (130)	0.3
Var × Age	Adip_M-image		0.030	0.075	0.40 (130)	0.7
Var × Age	Musc_F-image		0.32	0.11	2.85 (130)	0.005
Var × Age	Musc_M-image		0.17	0.11	1.52 (130)	0.1
Var × BMI	Adip_F-image		0.61	0.16	3.70 (130)	0.0003
Var × BMI	Adip_M-image		0.29	0.16	1.84 (130)	0.07
Var × BMI	Musc_F-image		−0.023	0.23	−0.10 (130)	0.9
Var × BMI	Musc_M-image		0.22	0.23	0.94 (130)	0.3
Var × DMS	Adip_F-image		0.16	0.064	2.55 (130)	0.01
Var × DMS	Adip_M-image		0.10	0.060	1.72 (130)	0.09
Var × DMS	Musc_F-image		0.025	0.090	0.28 (130)	0.78
Var × DMS	Musc_M-image		0.18	0.090	2.03 (130)	0.04
Var × SATAQ athl	Adip_F-image		−0.66	0.14	−4.63 (130)	<0.0001
Var × SATAQ athl	Adip_M-image		−0.40	0.14	−2.97 (130)	0.004
Var × SATAQ athl	Musc_F-image		0.55	0.20	2.70 (130)	0.008
Var × SATAQ athl	Musc_M-image		0.65	0.20	3.18 (130)	0.002
Random Effects	*Z* value	*p* value				
Adip_F-image variance	55.32	<0.0001				
Adip_M-image variance	49.23	<0.0001				
Musc_F-image variance	109.97	<0.0001				
Musc_M-image variance	110.66	<0.0001				

Adip_F-image, adipose in female stimuli; Adip_M-image, adipose in male stimuli; Musc_F-image, muscle in female stimuli; Musc_M-image, muscle in male stimuli.

**Figure 5:**
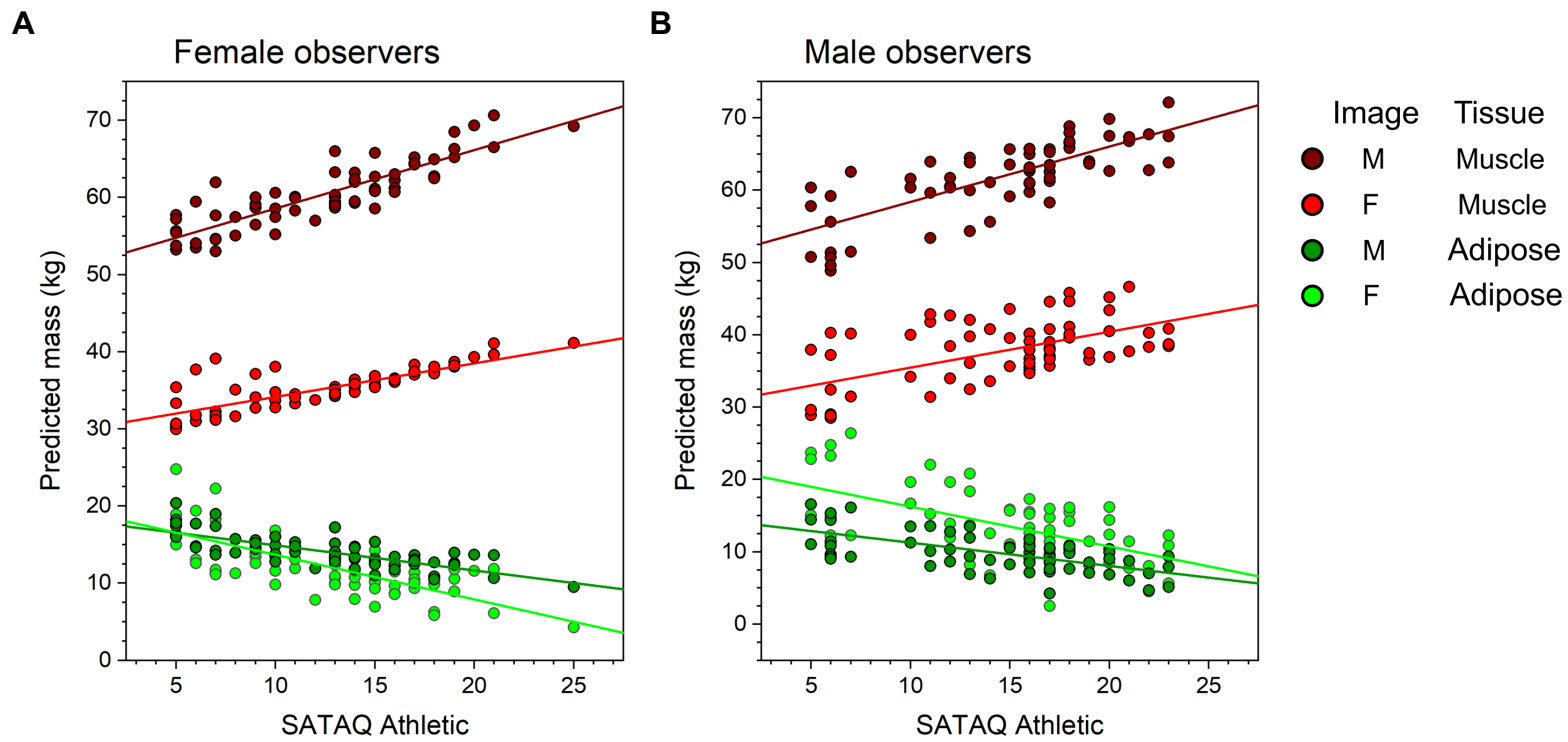
Scatter plots of muscle and adipose mass predicted by the multivariate multiple regression model reported in Table 5, plotted as a function of SATAQ athletic, and shown separately for **(A)**, female and **(B)**, male observers.

**Figure 6:**
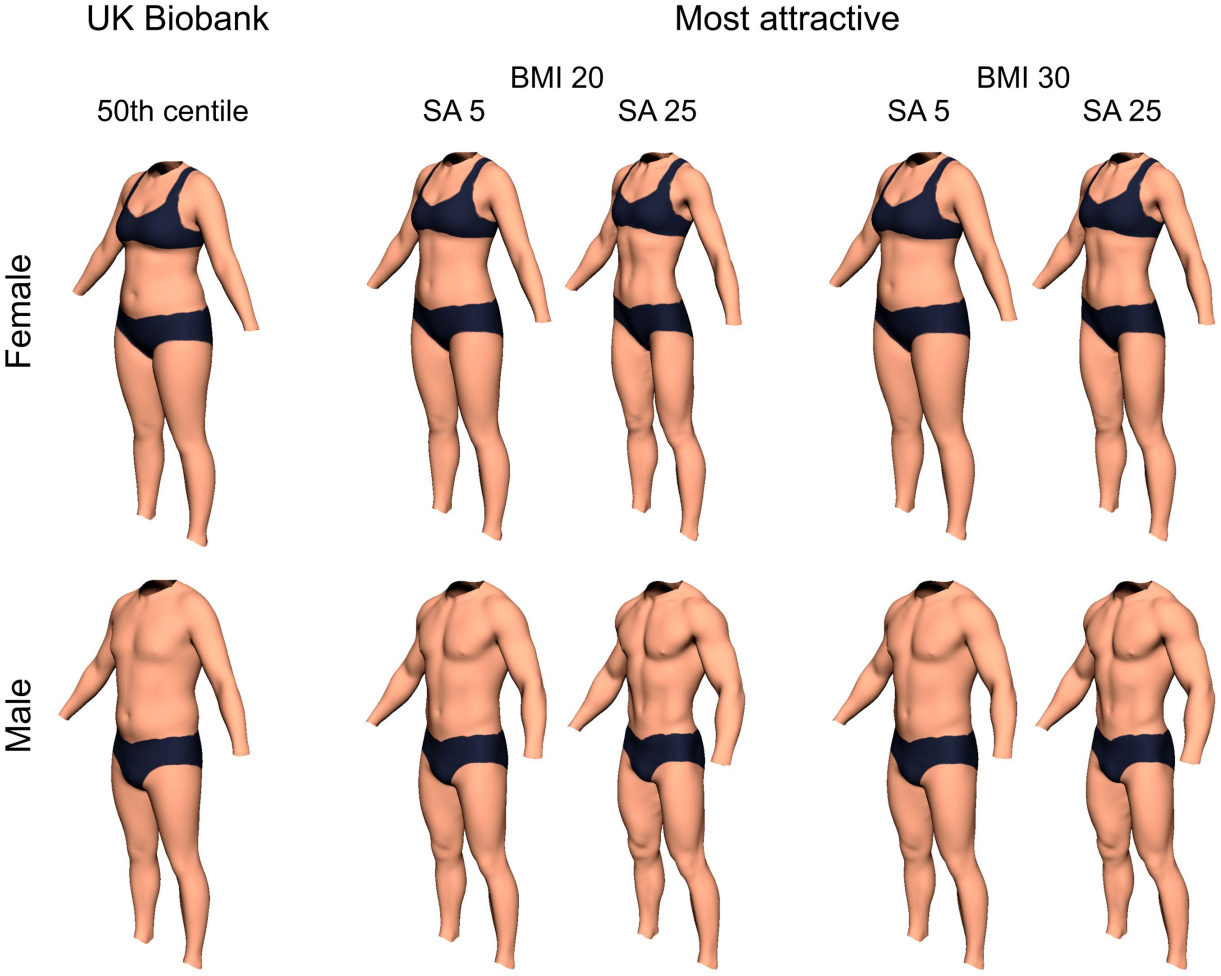
Illustrations of male and female bodies corresponding to the UK Biobank 50th centile, and for observers with BMIs of 20 and 30 respectively, divided further according to their SATAQ athletic (SA) scores of 5 (minimum) and 25 (maximum) respectively. Using the modelling technique of Maalin et al. (2021), set to the nearest 1 kg increment, the male UK Biobank body has a composition of Adipose 19.0 kg and Muscle of 37.0 kg. When the observer has a BMI of 20, the male SATAQ 5 body has Adipose of 14.0 kg and Muscle 55.0 kg, and the male SATAQ 25 body has Adipose of 6.0 kg and Muscle of 68.0 kg. When the observer has a BMI of 30, the male SATAQ 5 body has Adipose of 17.0 kg and Muscle 57.0 kg, and the male SATAQ 25 body has Adipose of 9.0 kg and Muscle of 70.0 kg. The Female UK Biobank body has Adipose 22.0 kg and Muscle 24.0 kg. When the observer has a BMI of 20, the female SATAQ 5 body has Adipose of 16.0 kg and Muscle 32.0 kg, and the female SATAQ 25 body has Adipose of 3.0 kg and Muscle of 43.0 kg. When the observer has a BMI of 30, the female SATAQ 5 body has Adipose of 23.0 kg and Muscle 32.0 kg, and the female SATAQ 25 body has Adipose of 9.0 kg and Muscle of 43.0 kg.

In addition, we computed a number of multivariate tests. Pillai’s Trace showed a statistically significant main effect of body composition (*V* = 0.22, *F*(2, 123) = 17.06, *p* < 0.0001): across all images, peak attractiveness was associated with higher muscle mass (*M* = 48.88 kg, SE = 1.01) than adipose mass (*M* = 12.54 kg, SE = 0.51). We found a significant two-way interaction between body composition and rater sex (*V* = 0.060, *F*(2, 123) = 3.93, *p* = 0.02): i.e., the images men found most attractive had more muscle (*M* = 49.85 kg, SE = 1.42) than those that women did (*M* = 47.84 kg, SE = 1.45). However, the images women found most attractive had more adipose (*M* = 13.15 kg, SE = 0.71) than those that men did (*M* = 11.97 kg, SE = 0.72). In addition, we found significant two-way interactions between body composition and age [*V* = 0.065, *F*(2, 123) = 4.25, *p* = 0.02], BMI [*V* = 0.070, *F*(2, 123) = 4.63, *p* = 0.01], DMS [*V* = 0.056, *F*(2, 123) = 3.65, *p* = 0.03], and SATAQ Athletic [*V* = 0.21, *F*(2, 123) = 16.60, *p* < 0.0001]. We also found significant three-way interactions between body composition, image sex, and rater sex [*V* = 0.072, *F*(2, 123) = 4.76, *p* = 0.01] as well as between body composition, image sex, and BMI [*V* = 0.063, *F*(2, 123) = 4.12, *p* = 0.02]. The most striking overall finding was that the more that observers’ internalized information about having an “athletic” body, the more they found high muscle, low body fat bodies to be most attractive. These interrelationships for SATAQ Athletic are further illustrated by the scatterplots in Figure 5. Moreover, we used the model illustrated in Table 5 to compute the LSMEAN adipose and muscle mass for men and women at the minimum (5) and maximum (25) SATAQ Athletic scores, for raters at BMIs of 20 and 30, respectively. These bodies representing the raters’ “most attractive” choices, together with the body shapes represented by the UK Biobank 50th centiles, are illustrated in Figure 6.

## Discussion of study 2

The locations for most attractive male and female bodies in body composition space, on average, appear to be in good agreement between Studies 1 and 2. This suggests good convergence between the two measurement methods, using the same stimulus set. Figure 4 shows clearly that, on average, the most attractive male and female bodies have less adipose and substantially more muscle than the 50th centile images from the UK Biobank, consistent with participants internalizing both thinspiration and fitspiration concepts. There are also subtle, but significant differences with respect to how female and male raters judge images of men and women. On average, male raters preferred images of women with slightly more muscle and adipose than female raters. However, female raters preferred images of men with slightly more adipose and marginally less muscle than male raters. While these differences were statistically significant, they raise an important question of whether they would be visually perceptible. In short, in order to decide whether these differences in body composition are potentially meaningful, we would need to know whether men and women can reliably detect them perceptually. To do this, for any location in body composition space, we would need to measure the smallest difference in body composition that men and women can reliably detect; the so-called just noticeable difference or JND (Gescheider, 1997). For both photographs and 3D CGI models of women, we know that the JND for BMI increases systematically as bodies become larger, and this is consistent with Weber’s law (Cornelissen et al., 2016). Therefore, in order to adequately interpret the observed differences in body composition between male and female raters’ most attractive judgements, it would be of value to obtain commensurate JND measurements of body composition. If these differences do matter, they should equal or exceed their respective JNDs.

## General discussion

A key finding of this study, as shown by Table 5, Figures 5, 6, is how individual differences in SATAQ athletic scores influence both muscle and adipose mass in the images which are judged to be most attractive: as participants increasingly internalize the athletic/muscular ideal, so the body fat of the most attractive image reduces, and muscle mass increases. A similar effect holds for participants’ individual differences in their drive for muscularity (indexed by the DMS) and muscle mass in male images. Figure 6 shows direct visualizations of men and women’s bodies at the UK Biobank 50th centile, and for the minimum and maximum SATAQ athletic scores, further divided according to raters’ BMI (20 vs. 30). Qualitatively, we would suggest that the SA 5 images in Figure 6 (i.e., the SATAQ athletic minimum score) are not that far removed from the UK population 50th centile and are unlikely to be good candidates for thin/fitspiration ideals, especially for the woman. This distinction is even clearer in relation to raters’ BMI, the SA 5 images appear even further from thin/fitspiration ideals as BMI increases. By comparison, the SA 25 images in Figure 6 (i.e., the SATAQ athletic maximum score) are likely to be a much better fit to the thin/fitspiration ideal. Therefore, we argue that these results preclude the possibility that internalization of media, peer, and family pressure with respect to athletic ideals leads to a single, immutable, internal visual representation of this ideal; it is not a fixed entity. Instead, there seems to be a dose dependent effect of the SATAQ. A score of 5 on the SATAQ athletic subscale means that every answer to the 5 items is “I definitely disagree,” so participants have not internalized this ideal, and the body composition of the “most attractive” is not far removed from the population norm, particularly for raters with a higher BMI. At the opposite extreme, a score of 25 means that every answer to the same 5 items is “I definitely agree,” equating to maximum internalization, and the body composition of the “most attractive” has now morphed into a configuration consistent with the thin/fitspiration ideal. In other words, despite the ubiquitous presence of thin/fitspiration images in society, potentially coupled to social pressures to conform to them, an individual’s most attractive internal visual representation does not look like this unless they internalize it sufficiently.

In turn, this raises two important questions. First, how much internalization is enough to give rise to eating disordered pathology? To address this question, Schaefer et al. (2019) administered the EDE-Q and SATAQ-4 to 787 college women, who were classified as “healthy” or “eating disordered” according to established clinical cut-offs for the EDE-Q. They then applied receiver operating characteristic curve analyses to identify what score on the SATAQ-4 thin/low body fat subscale optimized the sensitivity and specificity for detecting eating disordered women. They found that a score of 3.78 (i.e., 18.9 as a raw score) yielded an optimal sensitivity of 0.81 together with a specificity of 0.64. If we make the assumption that similar numerical criteria might apply to the SATAQ-4 athletic/muscular subscale reported here, then a woman would need to have moved almost 70% of the way towards acquiring the SA 25 image in our data as her internal visual representation of “ideal”/“most attractive,” before exhibiting life altering changes in behavior.

The second important question is whether it is necessary and sufficient for *all* individuals to have acquired such an extreme internal visual representation of “ideal”/“most attractive,” in order to trigger eating disordered behavior. Or, are there other factors which can act either additively or multiplicatively together with thin/muscular internalization, to bring different individuals to the same tipping point? This is important because some etiological theories about the development of eating disorders propose multiple risk pathways (Thompson et al., 1999; Stice et al., 2001). To address this question, Stice et al. (2001) asked whether chronic exposure to thin models, rather than brief exposure to them in the laboratory, increased body dissatisfaction. To do this, they randomly assigned 219 adolescent girls to a 15-month fashion magazine subscription or a no-subscription condition, and tracked changes in thin-ideal internalization, body dissatisfaction, dieting, negative affect, or bulimic symptoms at baseline, 10 months, and 20 months. First, the authors confirmed that participants in the subscription condition read “Seventeen” magazine for ~ 21 h compared to ~ 15 h for the controls, a statistically significant advantage of ~ 6 h. However, despite this extra exposure, they could not find a main effect of experimental condition on any of the main outcome variables. Nevertheless, the authors did identify a subset of vulnerable adolescents, characterized by initial elevations in perceived pressure to be thin, body dissatisfaction, and deficits in social support, and these vulnerable individuals were adversely affected by exposure to the content of “Seventeen” magazine. More recently, Stice et al. (2011) carried out an eight-year follow-up study in which they used classification tree analysis to estimate cut-points for identifying adolescent girls at risk for future onset of threshold, subthreshold, and partial eating disorders. They found amplification effects (interactions) between high body dissatisfaction and high depressive symptoms, as well as low body dissatisfaction and high self-reported dieting, leading to increased risk of developing eating disorders. Therefore, longitudinal data from the community, rather than experimental data from the laboratory, is consistent with the idea that individuals who reach a tipping point towards eating disorders do so as a result of interactions between multiple risk factors.

Thus far, we have considered the consequences of internalizing information about athletic/muscular ideals on what men and women judge as most attractive. But what about those individuals who do not internalize such information (i.e., image SA 5, Figure 6). Where does their most attractive representation stem from? We propose that the “low SATAQ (low internalization) body” seems not too far removed in appearance and composition from the population median body based on the UK Biobank data, and by implication the population mean. There is some evidence from the face perception literature that an average face, one closer to the population mean, is consistently rated as being attractive (Rhodes et al., 2000; Valentine et al., 2004; Potter and Corneille, 2008). Two potential explanations have been proposed to explain this finding. The first is based on evolutionary psychology and suggests that the preference may be couched in a mate choice framework. In this context, facial averageness is suggested to signal greater genetic diversity, which in turn, may result in greater parasitic resistance, whereas deviation from average could signal potential genetic disorders (the “bad genes” hypothesis; Thornhill and Møller, 1997; Zebrowitz and Rhodes, 2004). Alternatively, the preference could arise through the way the visual system processes sensory information (Halberstadt and Rhodes, 2000; Rhodes, 2006). Average or prototypical faces and non-face stimuli are processed more rapidly and easily, and as a result, produce a more positive rating from observers (Reber et al., 1998; Winkielman et al., 2006; Trujillo et al., 2014). Either of these explanations could potentially explain the preference for a body size and shape closer to the population mean in participants who have not been significantly influenced by the media.

Alternatively, in choosing what appears to be a more “average” body, the participants may have been using a different set of criteria to those we have been discussing above. For example, they may be considering the body based on its functionality (i.e., how well it can do the things they want to do), rather than the cultural expectations which usually drive the narrative used to frame the size and shape of an ideal body (Alleva and Tylka, 2021).

### Gender differences in judgements of attractiveness

Evolutionary psychology theory predicts that a heterosexual individual will have an accurate idea of what the opposite gender considers attractive, to allow them to judge their own relative attractiveness with respect to their peer group, and match this with the attractiveness of a potential partner (Buss and Schmitt, 2019). If this theory is correct, then there should be no gender difference in the judgement of either female or male beauty, as both sexes should use the same selection criteria for estimating attractiveness in a particular gender. Our results are largely consistent with this prediction. Both genders prefer male bodies with high muscularity and low-adipose content, and both genders prefer female bodies with low adipose content and a significant amount of muscle. This is broadly consistent with previous studies which suggest the same attractiveness preferences for both genders (Coetzee et al., 2011; Stephen and Perera, 2014), although some studies have found small differences (Demarest and Allen, 2000; Bergstrom et al., 2004; Grossbard et al., 2011; Crossley et al., 2012). Like these latter studies, we did find subtle gender differences in how a particular sex is judged, but as discussed above, these are small enough for them to be questionable if they cannot be visually distinguished. The clear message from this study is that there is considerable agreement between the genders over what, on average, constitutes an attractive body.

### Limitations and future research

#### Most attractive versus ideal

In this study, we have assumed that “most attractive” and “ideal” can be treated as synonymous. To our knowledge, no-one to date has used the same stimulus set to verify this assumption precisely. Moreover, there is also the important question about which sort of ideal one is referring to. Here, we assume that “most attractive” corresponds to the ideal body shape represented in our current Western culture and is therefore really a reference to other men’s and women’s body shapes: a third-party reference frame. However, if a participant is asked to configure a 3D model or pick an image that corresponds to what they would ideally like to look like, this amounts to a first-person version of an ideal, and there are reasons for thinking that first and third person ideals, for men at least, may not be equivalent. For example, Mohamed et al. (2021) asked men to represent their own ideal body shape using an interactive 3D modelling tool and measured their performance on a number of psychometric tools including the SATAQ-3 and DMS. They then used Principal Components Analysis (PCA) together with multiple regression to map the relationship between 3D model structure and psychometric performance. In this way, the researchers could visualize the body shapes corresponding to the minimum and maximum scores on the SATAQ-3 and DMS scales. While it is clear that increasing psychometric scores were associated with a desire for reduced body fat, increased muscularity, and increased muscle tone, these effects were visually not as salient as the effects we have found in the current study. If it is true that these differences between studies cannot be attributable solely to the different measurement methods, then this does suggest that first- and third-party body ideals may not be the same, at least for men. For these reasons, new research is required to better characterize the relationships between “most-attractive” and the two different “ideals” for both men and women.

#### Independent variation of muscle and adipose content

We would argue that these studies are the first to use body stimuli which are calibrated directly based on 3D measures of body shape change as a function of measured body composition. Previous studies have used body scales which systematically vary combinations of muscle mass and adiposity in so-called somatomorphic matrices [e.g., (Cafri and Thompson, 2004; Talbot et al., 2019)]. However, it is unclear just how effective the calibration of these stimuli has been. In the first of these studies (Gruber et al., 1999), the starting point was a set of figures corresponding to particular fat-free mass indices (FFMIs) and body fat percentages (BF%). To achieve this, people with known FFMIs and BF%s determined by skinfold measurements with calipers, were photographed, and a graphic artist developed these images into drawings (Gruber et al., 1999). “Further validation was achieved by having experienced kinanthropists (i.e., experts at body composition assessment) review the images produced by the graphic artist, which resulted in an extensive process of revision until it was possible to reliably assign the correct FFMI and BF% to each image in the matrix” (Cafri and Thompson, 2004). The original somatomophic matrix was a set of 100 line drawn silhouettes (Gruber et al., 1999), which was subsequently simplified to a set of 34 line drawings (Cafri and Thompson, 2004), although this set shows poor reliability (Cafri et al., 2004). These hand-drawn bodies have been criticized for being of poor quality and providing limited information about musculature and tone (Talbot et al., 2019). To try and address this issue, Talbot et al. produced a CGI set of images based on this set of drawings with the addition of simulated muscle tone and shape, but there is no clear biometric foundation for these additions (i.e., these additions were not based on the appearance of bodies of a particular composition). Another approach has been to morph between bodies of high or low muscularity or adipose (Brierley et al., 2016). However, the very narrow range of muscle and adipose within the set of images in this study to morph between significantly limit the range of simulated change produced, and more importantly, this methodology will not produce fully independent change in muscle or adiposity.

We acknowledge that the stimulus set used here is not without limitations. First, the overall sample size of scanned bodies used by Maalin et al. (2021) would ideally be larger and cover a wider range of body composition. Second, the modelling method, again a combination of PCA and multiple regression, does not treat the three body compartments: skeletal structure, body fat, and skeletal muscle mass, entirely independently. As a consequence, there is a degree of covariation of skeletal structure as muscle and adipose mass change.

## Conclusion

The results from the two studies reported here show a clear preference, on average, by both genders for a male body with high muscle and low adipose, and a toned, low adipose female body. However, there are important differences between individuals in the precise position of their preferred body in the 2D body composition space. These differences seem to be driven by the degree to which the cultural ideal for muscularity/athleticism is internalized by an individual: the greater the internalization, the more extreme the muscle content and adipose reduction in the preferred ideal for both the same and opposite gender bodies.

## Data availability statement

The raw data supporting the conclusions of this article will be made available by the authors, without undue reservation.

## Ethics statement

The studies involving human participants were reviewed and approved by The Department of Psychology ethical committee at Northumbria University and the Business, Law and Social Sciences Faculty Academic Ethics Committee at Birmingham City University. The patients/participants provided their written informed consent to participate in this study.

## Author contributions

MT, BR, and PC designed the study. NM, SM, MT, and RK created the stimuli. KM programmed the studies. BR, NM, and SM collected data. PC, KM, and RK analyzed the data. MT and PC wrote the first draft of the manuscript. All authors contributed to the article and approved the submitted version.

## Conflict of interest

The authors declare that the research was conducted in the absence of any commercial or financial relationships that could be construed as a potential conflict of interest.

## Publisher’s note

All claims expressed in this article are solely those of the authors and do not necessarily represent those of their affiliated organizations, or those of the publisher, the editors and the reviewers. Any product that may be evaluated in this article, or claim that may be made by its manufacturer, is not guaranteed or endorsed by the publisher.
