# Supplementary figures and images for: The degree to which the cultural ideal is internalized predicts judgments of male and female physical attractiveness
Source: Front Psychol. 2022 Oct 19;13:980277. doi: 10.3389/fpsyg.2022.980277 (PMC9626828; doi:10.3389/fpsyg.2022.980277)

**Supplementary Material**


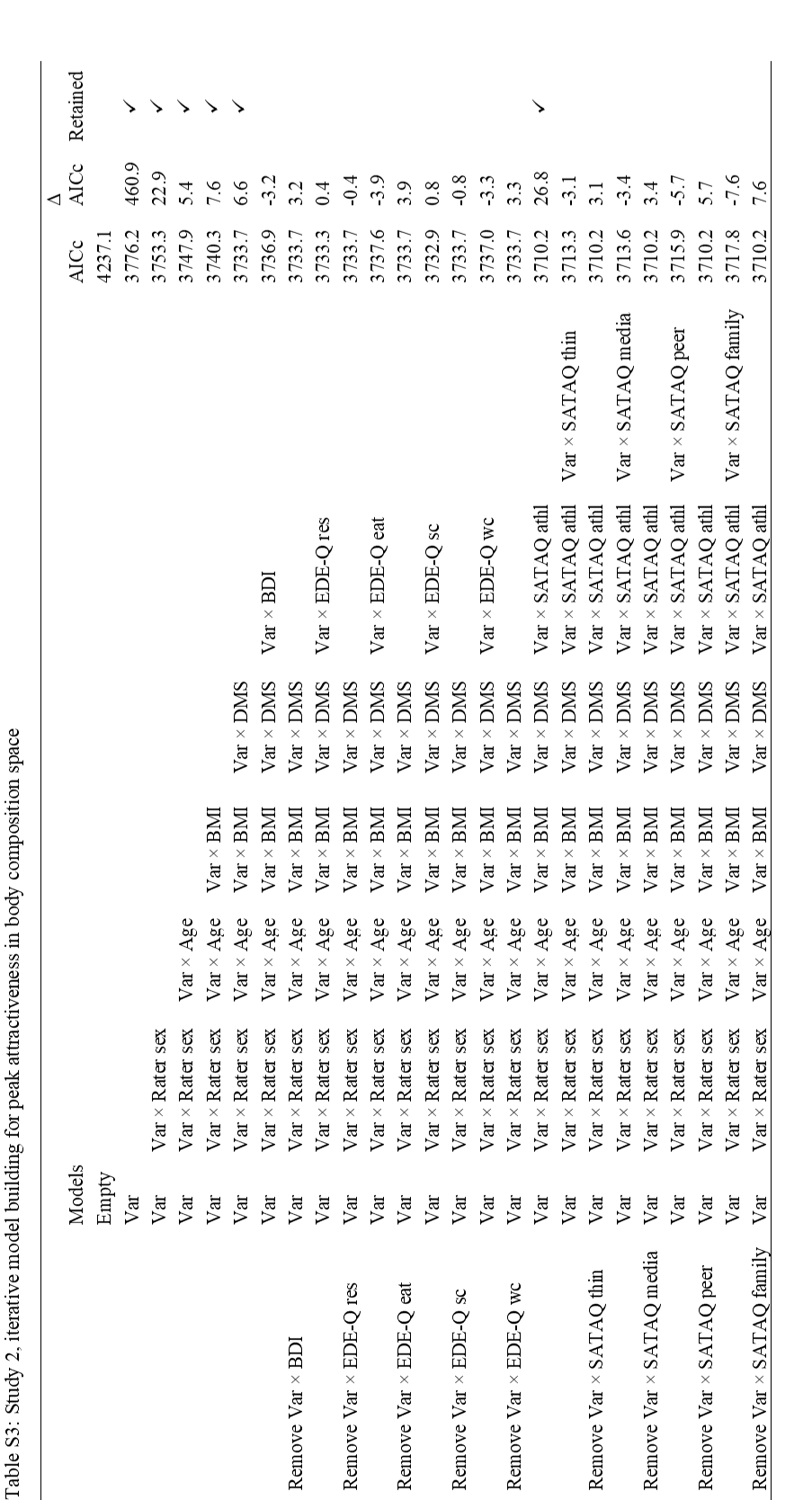

Supplement: Supplementary file 3 [file Table_3.docx]
